# Antenatal health promotion via short message service at a Midwife Obstetrics Unit in South Africa: a mixed methods study

**DOI:** 10.1186/1471-2393-14-284

**Published:** 2014-08-21

**Authors:** Yan Kwan Lau, Tali Cassidy, Damian Hacking, Kirsty Brittain, Hanne Jensen Haricharan, Marion Heap

**Affiliations:** Health and Human Rights Programme, School of Public Health and Family Medicine, University of Cape Town, Cape Town Observatory, 7925 South Africa

**Keywords:** Health education, Mobile health, Antenatal care

## Abstract

**Background:**

Adequate antenatal care is important to both the health of a pregnant woman and her unborn baby. Given South Africa’s high rate of cellphone penetration, mobile health interventions have been touted as a potentially powerful means to disseminate health information. This study aimed to increase antenatal health knowledge and awareness by disseminating text messages about clinic procedures at antenatal visits, and how to be healthy during pregnancy.

**Methods:**

Participants recruited were pregnant women attending a primary health care facility in Cape Town. A controlled clinical trial was carried out where the intervention group (n = 102) received text messages staggered according to the week of pregnancy at the time of recruitment. The control group (n = 104) received no text messages. These text messages contained antenatal health information, and were delivered in English, Xhosa or Afrikaans, according to the preference of each participant. A baseline knowledge questionnaire with nine questions was administered prior to the intervention. The same questionnaire was used with added health-related behaviour questions for the intervention group at exit. A modified intention-to-treat analysis was done. To compare the control and intervention group’s knowledge, Fisher’s exact tests and two-sample t-tests tests were carried out for binary and continuous outcomes, respectively. A focus group of seven participants from the intervention group was then conducted to gain more insight into how the text messages were perceived.

**Results:**

There was substantial loss to follow-up during the study with only 57% of the participants retained at exit. No statistically significant difference was detected between the control and intervention group in any of the nine knowledge questions at exit (all p > 0.05). Responses from the focus group indicated that the text messages acted as a welcome reminder and a source of positive motivation, and were perceived as extended care from the health care provider.

**Conclusions:**

While the intervention failed to improve antenatal health knowledge, evidence from self-reported behaviour and the focus group suggests that text messages have the potential to motivate change in health-seeking behaviour. One should be mindful of loss to follow-up when rolling out mobile health interventions in developing country settings.

**Trial registration:**

Pan African Clinical Trials Registry PACTR201406000841188. Registered 3 June 2014.

**Electronic supplementary material:**

The online version of this article (doi:10.1186/1471-2393-14-284) contains supplementary material, which is available to authorized users.

## Background

South Africa has provided free comprehensive antenatal care to all pregnant women since July 1994 [1]. Despite this, there is still a high prevalence of late initiation of antenatal care and a low attendance of the recommended number of visits according to national guidelines [2–4]. Consequently, the effectiveness of antenatal care to prevent complications from developing during pregnancy and childbirth is limited; the most recent *Saving Mothers 2008–2010* report indicates that 23.5% of assessable maternal deaths are avertable with sufficient antenatal care [5].

Socioeconomic factors, pregnancy confirmation time, health condition of pregnant women, and the availability, affordability and acceptability of antenatal care are all barriers to its utilisation [4, 6–8]. However, these do not fully explain the patterns of utilisation: inadequate utilisation of antenatal care is also due to a lack of perceived benefit of antenatal care by pregnant women, as well as a lack of understanding regarding how it can address potential threats to the health of both the mother and the child [6, 7]. It is in this context that the intervention of this study is conceived: to provide timely, bite-size information regarding procedures at antenatal clinic visits and, more generally, how to be healthy during pregnancy. In turn, being better informed could motivate pregnant women to attend clinics as prescribed and potentially improve health-related behaviours.

Mobile health or “mHealth” - the application of mobile technology to address healthcare issues - has become widespread, particularly in developing countries, as mobile networks can overcome lack of infrastructure such as roads, electricity and fixed line internet [9]. Applications of mHealth include: communicating health information, promotion of adherence to medication, and appointment reminders [10–12]. In particular, short message service (SMS), a text messaging feature available to all cellphones, limited usually to 160 characters, could be an attractive mHealth application to bridge the information gap in healthcare [10–12]. Three primary reasons for the enthusiasm are: high cellphone penetration across all socioeconomic groups, relatively low cost of SMS, and almost real-time delivery of information [10–12]. In South Africa, the mobile penetration was as high as 123% in 2012 [13]. Further, approximately 75% of those who are 15 years or older in low-income groups own a cellphone [14], and up to 69% of cellphone owners prefer sending SMS to calling, as the former is less expensive [15]. There have been various pilot studies in the US such as text4baby [16] and one carried out at Lynn Community Health Center [17]. However, neither measured antenatal health knowledge as an outcome and both studies used survey methods only. While there have been many SMS interventions carried out in developing country settings, few have been evaluated [12, 18, 19]. There have been even fewer interventions evaluated related to antenatal health knowledge in a resource-limited setting [20, 21].

The primary objective of this study was to evaluate whether antenatal health information disseminated via SMS would increase health knowledge in pregnant women. To this end, a controlled clinical trial was conducted and the antenatal health knowledge between the control and intervention group was compared at exit. In addition, the acceptance of SMSes as a medium of transferring health knowledge was evaluated, as well as their impact on self-reported health-related behaviours among those who received the intervention. This was evaluated via a focus group discussion with seven participants from the intervention group.

## Methods

### Population and setting

This study was conducted at a primary health care facility in the planning District F Mitchells Plain/Khayelitsha, Cape Town. This district is characterised by a young population (median age: 23 years), high unemployment (30.9%) and a high proportion of informal dwellings (34.9%) in comparison to other districts in Cape Town [22]. Participants recruited were pregnant women: i) attending their first antenatal clinic visit at the health care facility, ii) over 18 years of age, iii) with access to a cellphone number. At recruitment, trained fieldworkers handed out an information pamphlet available in the three local languages of Xhosa, English and Afrikaans, which explained the study. Fieldworkers were also present to clarify any questions that arose. Women who expressed interest in participating signed a consent form in a private room in the health facility, which was read out to them by the fieldworkers. The study was approved by the University of Cape Town’s Health Science Faculty Human Research Ethics Committee (Approval number 044/2011, 12 April 2011). Further, the reporting of this study complied with the CONSORT checklist (see Additional file 1).

### Antenatal SMS - the intervention

In consultation with the midwives and health promoters at the health care facility where the study was conducted, the type of information and a list of topics that were shared with their clients by trimester were established. Midwives agreed that SMS reminders could potentially be useful to women who were forced to take in a lot of new information on their first clinic visit. The information was further confirmed by visiting the health care facility on several occasions. A list of SMSes was then created in English based on the ascertained information. The appropriateness of the SMSes was verified by: the midwives at the health care facility, a health promotion specialist at the School of Public Health and Family Medicine at the University of Cape Town, a specialist obstetrician, and a specialist on creating SMSes from an organisation that is involved in mHealth applications. The SMSes were then translated into Afrikaans and Xhosa, two widely spoken languages in Cape Town. Participants could then choose the language in which they would like to receive the SMSes. Given the challenges of translating the same meaning into Xhosa in 160 characters, those SMSes were back translated to double check the translations. All SMSes (in all three languages) were then tested among the fieldworkers and researchers. The total number of SMSes created per language was 101. As health information given by health care workers and needed by pregnant women differs by trimester, the SMSes were also tailored according to trimester. Therefore, the sending of SMSes was staggered according to the participant’s week of pregnancy at the time of recruitment. For example, for a woman booking at 10 weeks, there were sixteen weeks before the third trimester and SMSes were sent three to four times a week. For a woman recruited at 19 weeks, there were eight weeks left of second trimester. One SMS was sent daily. By the third trimester, those that had booked early and those that had booked later were receiving SMSes at the same rate of three to four per week. For the exact distribution of SMSes by content and week of pregnancy at the time of recruitment, please see Additional file 2. The themes covered by trimester can be seen from Additional file 3.

### Sample size, sampling and study design

**Figure 1:**
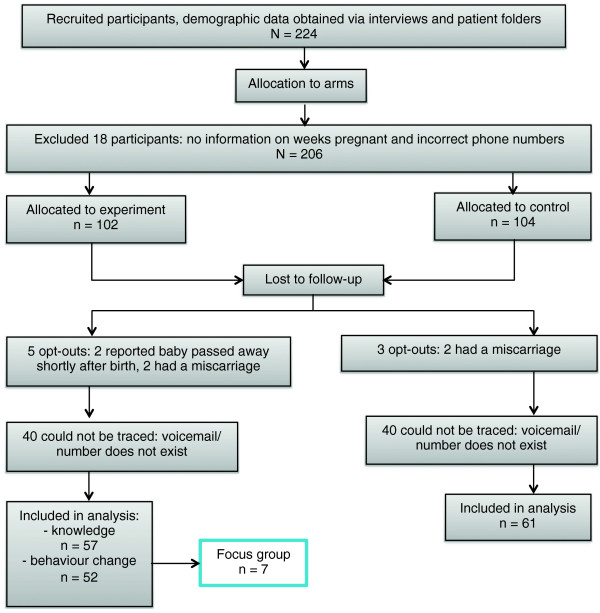
**Participant diagram.**

A sample size of 100 participants per intervention arm was calculated. Mean knowledge scores for all three parts – general antenatal health, clinic procedures, and the total – were assumed to be half of the maximum of each component i.e. 6, 3, and 9 respectively. The standard deviation of each were further assumed to be 10% of the point total for each component. These assumptions were made in the absence of baseline levels of knowledge among women in our study population prior to carrying out the study. A total sample size of 200 gives statistical power of over 95% at the 5% level of significance. Convenience sampling was performed where participants attending antenatal visits at the health care facility were recruited over a period of six weeks from 16 July 2012 to 23 August 2012. Participants were interviewed at the facility on the day of recruitment, and antenatal knowledge data were collected by a form on a cellphone, which was uploaded by the fieldworkers at the end of the day to a database. In particular, data collection was done using an mHealth solution provided by Cell-Life, based on openXdata. The database is hosted by a server where the data were sent from the cellphone via HTTPS. The database is password protected and was only available to the authors of the paper. Each participant’s cellphone number, date of birth and gestational age were also collected. An initial 224 participants were recruited to counter for potential loss to follow-up. From the database, participants were alternately assigned into intervention and control groups, and then stratified by language group (Xhosa, English and Afrikaans) and week of pregnancy. This was done by the principal investigator (MH) who was not involved with the recruitment. 18 participants were excluded as there were either incorrect phone numbers or no information regarding gestational age, resulting in 102 participants in the intervention group and 104 in the control group at baseline. At exit, the number of participants in these groups was 57 and 61 respectively due to loss to follow-up. See Figure 1 for the participant flow of the study.

### Baseline assessment

Week of pregnancy was obtained from patient folders at the health care facility after each participant’s appointment. Demographic data – date of birth, marital status, education, employment status, monthly income and number of children – were collected when the participants were enrolled into the study (see Table 1).

**Table 1:** **Baseline characteristics of participants (N = 206)**

	Intervention (n = 102)	Control (n = 104)	p-value
	Mean or n (SD or%)		
Age (mean)	27.61 (5.76)	26.28 (5.77)	0.072
**Marital status** (n)	0.945		
Married	42 (41.18)	45 (43.27)	
Single living with family	43 (42.16)	39 (37.50)	
Single living with partner	14 (13.73)	16 (15.38)	
Single living with people not family	3 (2.94)	3 (50.00)	
Widow	0 (0.00)	1 (2.88)	
**Employment status** (n)	0.485		
Employed	48 (47.06)	54 (51.92)	
Unemployed	54 (52.94)	50 (48.08)	
**Monthly income** (n)	0.677		
None	45 (44.11)	46 (44.23)	
Social grant	6 (5.88)	3 (2.88)	
< R4000	44 (43.14)	49 (47.12)	
R4000 - R10000	7 (6.86)	5 (4.81)	
> R10000 (US$1 = R8.20 in 2012)	0 (0.00)	1 (0.96)	
**Education** (n)	0.456		
Primary school	1 (0.98)	2 (1.92)	
Some high school	55 (53.92)	56 (53.85)	
Diploma	3 (2.94)	0 (0.00)	
Completed high school	43 (42.16)	46 (44.23)	
**Weeks pregnant** (n)	0.782		
1st trimester	35 (34.31)	32 (30.77)	
2nd trimester	67 (65.69)	72 (69.23)	
**Number of children** (n)	0.091		
0	24 (23.53)	39 (37.50)	
1 - 2	66 (64.71)	56 (53.85)	
> = 3	12 (11.76)	9 (8.65)	
**Language chosen** (n)	0.908		
English	82 (80.39)	86 (82.69)	
Afrikaans	7 (6.86)	6 (5.77)	
Xhosa	13 (12.75)	12 (11.54)	

Prior to the start of the SMS campaign, a questionnaire was administered to all participants in order to measure their knowledge of antenatal health at baseline. The questionnaire included nine multiple choice questions, five of which assessed knowledge regarding general antenatal health, and four which pertained to clinic procedures (see Table 2, full questionnaire in Additional file 4). Each correct answer was awarded one point. In cases where there were more than one correct answer, an incorrect answer chosen would result in one point being deducted. This was to control for participants choosing all possible options due to lack of knowledge. The minimum score for any question was zero.

**Table 2:** **Knowledge levels at exit (N = 118)**

	Intervention (n = 57)	Control (n = 61)	p-value
	[95% CI] or (proportion of correct answers)		
**Mean clinic procedures score (max = 6)**	3.05 [2.80-3.31]	3.05 [2.77-3.33]	0.99
1. Is it important to attend clinic when you are pregnant?^*^	57 (100)	61 (100)	-
2. Why do the nurses test the blood?	0.61 [0.39-0.84]	0.66 [0.46-0.85]	0.39
3. Should you ask for the results of your pap smear?^*^	51 (89.47)	53 (86.89)	0.44
4. Why do the nurses test the urine and blood pressure every visit?^*^	31 (54.39)	32 (52.46)	0.97
**Mean general antenatal health score (max = 12)**	7.14 [6.44-7.84]	7.34 [6.78-7.90]	0.65
5. How can you stay healthy during pregnancy?	2.28 [1.95-2.61]	2.38 [2.08-2.68]	0.66
6. Why should you take folic acid during your pregnancy?^*^	23 (40.35)	28 (45.90)	0.49
7. How do drugs and alcohol affect the baby growing in the womb?	1.53 [1.37-1.69]	1.49 [1.34-1.64]	0.75
8. Should you seek medical help outside your appointments?	1.37 [1.17-1.57]	1.36 [1.14-1.58]	0.96
9. What are the signs of labour?	1.56 [1.31-1.81]	1.66 [1.40-1.91]	0.60
**Mean overall score**	10.19 [9.80-10.58]	10.39 [10.03-10.72]	0.69

^*^Only one correct answer hence the number of correct answers and in parentheses, the proportion of correct answers, are reported.

An end-of-campaign date was calculated for each woman where a full term pregnancy is assumed to have occurred after 37 weeks. She was interviewed by phone as soon after this date as possible. However, because some women were easier to contact than others, the date between the end of the campaign and the exit interview was not consistent for each participant. At this point, all participants’ knowledge levels were re-evaluated using the same questionnaire. Again, a score was assigned to each question. In addition, a score for each dimension – general antenatal health knowledge and clinic procedures – was calculated for both baseline and exit knowledge. The maximum scores for each dimension were 12 and 6 respectively. Self-reported behavioural changes (see Table 3) and data regarding other sources of antenatal information were also collected from those who reported that they had received SMSes in the intervention group. The latter involved asking: “From where did you get information about pregnancy?”, “Which source had the most impact on you?”, “Did you find the SMSes useful?”, and “Did the SMSes give you new information that made you change your lifestyle or manage your pregnancy different?”

**Table 3:** **Self-reported health behaviours of participants in the intervention arm (N = 52)**

Question	n (%)			
	Yes	No	Don’t know	Not applicable
*Did you miss more than two clinic appointments?*	4 (7.69)	48 (92.31)	-	-
*Did you make sure you got the results of your pap smear?*	34 (65.38)	9 (17.31)	2 (3.85)	7 (13.46)
*Did you take folic acid and iron during your pregnancy?*	48 (92.31)	3 (5.77)	1 (1.92)	-
*Did you drink alcohol during your pregnancy?*	1 (1.92)	51 (98.08)	-	-
*Did you take drugs (such as tik, dagga, mandrax, heroin, cocaine etc.) during your pregnancy?*	-	52 (100)	-	-
*Did you smoke (tobacco) during your pregnancy?*	12 (23.08)	40 (76.92)		
*Did you eat healthily during your pregnancy?*	47 (90.38)	4 (7.69)	1 (1.92)	-
*Did you exercise/made sure you stayed fit during your pregnancy?*	51 (98.08)	1 (1.92)	-	-
*Did you take any prescription medication without discussing it with your health care provider during your pregnancy?*	5 (9.62)	47 (90.38)	-	-

### Focus group

Following the exit questionnaire, a focus group of seven participants from the intervention group was conducted in order to gain more insight into the participants’ experiences of the SMS campaign, given the findings of the questions regarding self-reported behavioural change. Similar to the controlled trial, convenience sampling was done to recruit participants to the focus group. To be eligible for the focus group, participants had to have received over 70% of the SMSes, and be willing to discuss the SMS campaign. The focus group was conducted by one investigator, observed by one research assistant, and recorded by a dictaphone and written notes. The recordings were subsequently transcribed and were analysed using thematic analysis. An Afrikaans translator was also present during the focus group for those participants whose first language was not English. The interview guide can be found under Additional file 5.

### Data analyses

A modified intention-to-treat-analysis was done for knowledge outcomes i.e. those in the intervention arm who reported not having received SMSes were still included in the analysis of the knowledge scores. Differences in knowledge between the intervention and control group for continuous outcomes (i.e. questions with multiple answers and knowledge score subtotals) were examined using two-sample t-tests. The assumption of equal variance was verified in each of these instances. Fisher’s exact test (as opposed to chi-squared test) was used to assess questions with multiple responses but only one correct answer given the unequal distribution of the responses per question. Frequency statistics were reported for self-reported behavioural change and importance of sources of antenatal health information. Note that five participants from the intervention group reported that they had not received any SMSes; these participants were excluded from analysis of the behavioural change segment. All statistical analyses were conducted using Stata 11.2 [23].

## Results

### Baseline characteristics

Participants at baseline had a mean age of 26 years, and were mostly married or, single and living with family. Approximately half were unemployed, with most reporting a monthly income of R4000 (US$ 487.80 in 2012) or less followed by no income, and most have completed some high school or more. Approximately two-thirds attended their first antenatal visit during their second trimester, and almost 70% reported to have at least one child. As can be seen from Table 1, the control and intervention groups were comparable at baseline as they were not significantly different from each other in any of the variables. Regarding antenatal knowledge at baseline, all of the responses between the two groups were comparable at baseline except for question 9 which asked, “What are the signs of labour?” (results not shown).

### Knowledge and behaviour

There was a large amount of loss to follow-up (LTFU) during the study, as only 57% (N = 118) of the participants were retained from baseline. It was found, however, that there was no differential LTFU. From Table 2, it can be seen that no statistically significant difference in scores was detected in any of the nine questions between the intervention and control group. Given these results, it is interesting to compare with the responses given in Table 3. The intervention group’s overall mean score was 10.19 out of a maximum of 18, indicating that the participants only answered about 55% of the questions correctly. When asked behaviour questions, the participants in the intervention group reported fairly healthy behaviours during pregnancy, despite a relatively low mean knowledge score (see Table 3). In addition, four questions were asked of the intervention group about the sources from which they received information regarding antenatal health. The main reported source of information about pregnancy was from SMSes (98%) and then health promoters (90%). The SMSes were also reported to have had the most impact (88%) compared to health promoters (33%) and friends/family/colleagues (15%). It is interesting to note that while the vast majority of the 52 participants reported that the SMSes were useful (98%) and had an influence in changing their lifestyles (96%), their knowledge level did not seem better than those who did not receive SMSes. This served as the starting point for the focus group conducted.

### Focus group

Owing to what seems to be an incongruity between knowledge and self-reported healthy behaviours, the primary aim of the focus group was to explore the impact that the SMS campaign had in terms of how the SMSes had influenced healthy behaviours.

The participants argued that they had improved health-related behaviours as a result of the SMSes. They reported that they adhered better to the treatment of sexually transmitted infections, had started eating healthier and exercising, took folic acid and vitamins, and attended the clinic regularly. The campaign reportedly made the participants feel more confident, and more engaged in their pregnancy. A participant mentioned that she felt she was “on the right route” and motivated “to do the right thing.” Two general themes that emerged from the focus group were the trustworthiness of the information from a “caring” campaign, and how the SMSes functioned as a “friendly reminder”.

### Trustworthy information

The participants were asked how they perceived the information from the SMS campaign.

“*It’s like it’s the Department of Health, ne? So it’s coming straight from the people who know what they are talking about*”.

“… *came from educated people*”.

These statements suggest that the SMSes were seen as more trustworthy than the other sources of information due to the perceived authority of the SMSes, which were associated with official sources. The appreciation for the “caring” element can be seen from the following:

“*There was someone there willing to help you through the real pregnancy*”.

“*And it’s also good because it just shows that there are people out there that’s taking time to send you that SMS*”.

### Friendly reminder

“*The SMSes stay on your phone. They can stay on your phone, and you can just go back and read it again… it’s like a constant reminder”.*

The SMSes were said to be “short and sweet”. Participants noted that the SMSes were easily accessible and that they did not need to search the internet or read books in order to access information, which was important as participants mentioned that they were “tired and busy”. The other advantage reported was that the SMSes were also easily retrievable as they stay on the cellphone, unlike the health talks at the clinic which were easily forgotten. Participants also stated that they liked the repetition and reminding inherent in repeated SMSes on the same topic. Overall, there was consensus among participants about the value and impact of the SMS campaign with no deviant cases.

## Discussion

Analysis of the quantitative results showed that there was no difference in antenatal health knowledge levels at exit between the control and intervention group (all p > 0.05). This could have several explanations. Firstly, the mean message delivery rate to those in the intervention group was only 70%. This means that not all participants received all 101 SMSes due to technical failures on the part of the cellular service provider, and/or participants did not have their phones switched on for over 48 hours. Secondly, based on the experience of fieldwork and data collection at exit, it was discovered that phone sharing between participants with members of their family or a neighbour was not uncommon. The implication is that even if messages were successfully delivered, the participant may not have read them.

A high level of beneficial and positive health-related behaviours during pregnancy among participants in the intervention group was reported. This is comparable to other health promotion SMS campaigns where participants reported eating healthier [16]. The focus group results suggested that the SMS campaign played an important role in these behaviours. Results presented from the focus group reveal that although information may already be known, it was re-enforced in an easy, accessible way through the SMSes. Although we failed to find a significant change in knowledge, one should not discount the reassurance that reminders from a perceived trustworthy source reportedly gave to participants who were “tired and busy”. This was a similar finding to another pilot study by Pelletier and colleagues [17], where participants said “it made me feel supported by the team” (p. 38). Applying the Stage Theory of Health Behaviour/Transtheoretical Model [24], there seems to be evidence that the SMSes could play a beneficial role as a cue to advance people from the *Contemplation* and/or *Preparation* stage to the *Action* stage. There is reason to believe that the participants were in the *Preparation* stage at baseline, given that they were present at the clinic for their first antenatal visit and volunteered to be part of the study.

### Limitations

There was substantial loss to follow-up during the study. Even though differential loss to follow-up was not detected amongst the demographic variables that were collected, the low retention rate of 57% could compromise the validity of the findings presented. Further, healthy behaviours were self-reported, and responses could have been unduly influenced by social desirability bias. It was difficult to include objective measures of behavioural change in this study i.e. whether a participant was eating a healthier diet. It was also not clear whether these self-reported healthy behaviours would be maintained. Saturation was reached amongst the members of the focus group, but given that only one focus group was conducted possible themes may not have been uncovered. A further limitation of the study is that the focus group might have only consisted of very motivated individuals who were happy with the SMS campaign i.e. volunteer bias could be present, and the results from the focus group may thus not be generalisable.

## Conclusions

This study provided insight into a text-message based mHealth intervention carried out among pregnant women in Cape Town, South Africa. While the SMS campaign did not show evidence of improving antenatal health knowledge, there is some evidence that it promoted healthy behaviours during pregnancy. Future research should examine whether the self-reported behaviour change is maintained over time, and develop objective measures of behaviour change. Governments and organisations can take heed of the lessons learnt from this study when considering the use of SMSes as a means to promote health education in a low-resource setting.

## Electronic supplementary material

Additional file 1: **CONSORT checklist.** (XLS 414 KB)

Additional file 2: **Distribution of SMSes by content and week of pregnancy.** (DOCX 86 KB)

Additional file 3: **Health themes covered in the SMSes by trimester.** (DOC 68 KB)

Additional file 4: **Exit questionnaire.** (DOCX 20 KB)

Additional file 5: **Focus group question guide.** (DOC 218 KB)

## Abbreviations

SMS: Short message service.
