# Supplementary material for: Antenatal health promotion via short message service at a Midwife Obstetrics Unit in South Africa: a mixed methods study
Source: BMC Pregnancy Childbirth. 2014 Aug 21;14:284. doi: 10.1186/1471-2393-14-284 (PMC4158091; doi:10.1186/1471-2393-14-284)
Supplement: Supplementary file 2 — Additional file 2: Distribution of SMSes by content and week of pregnancy. (DOCX 86 KB) [file 12884_2014_1164_MOESM2_ESM.docx]

SMS THEMES BY TRIMESTER

**First and Second Trimester: Getting Ready and healthy living during pregnancy**

## Theme 1: Congratulations, welcome, rules and 1^st^ tip

Theme 2: Pregnancy is not an illness, but check-ups are important

## Theme 3: When to contact your clinic, take care of yourself

## Theme 4: Emotional well-being of the mother during pregnancy

## Theme 5: Sexually transmitted diseases

# Theme 6: Understanding tests

## Theme 7 (two weeks): Healthy living: Alcohol and drugs

## Theme 8: Healthy living: smoking

## Theme 9: Healthy living: Medicine

## Theme 10. Healthy living: Folic acid and vitamins

## Theme 11. Baby beginning to move + mum beginning to gain weight

## Theme 12. Healthy living, exercise and sex

## Theme 13: When to see clinic, emergency and pre-eclampsia

## Theme 14: Healthy living: Food to eat

**Third trimester: getting ready for birth and mothering**

## Theme 15: HIV

## Theme 16: Baby getting bigger, start to slow down

## Theme 17: Breastfeeding

## Theme 18: Breastfeeding, continued

## Theme 19: Continued healthy living, emotional well-being, attend clinics, pre-eclampsia.

## Theme 20: Make sure you have support

## Theme 21: Be ready for labour

## Theme 22: Packing your bag

## Theme 23: Take care of yourself after baby is born, postnatal depression

## Theme 24: Register your baby + family planning

## Theme 25: Take care of your baby, attend clinic and immunisation

## Theme 26: Good bye and good luck. Take care of yourself and your baby
