# Supplementary material for: Antenatal health promotion via short message service at a Midwife Obstetrics Unit in South Africa: a mixed methods study
Source: BMC Pregnancy Childbirth. 2014 Aug 21;14:284. doi: 10.1186/1471-2393-14-284 (PMC4158091; doi:10.1186/1471-2393-14-284)
Supplement: Supplementary file 3 — Additional file 3: Health themes covered in the SMSes by trimester. (DOC 68 KB) [file 12884_2014_1164_MOESM3_ESM.doc]

ANTENATAL CARE SMS CAMPAIGN: EXIT QUESTIONNAIRE

1. **Is it important to attend clinic when you are pregnant? [maximum score: 1]**
   1. Yes [1]
   2. No, not if you are healthy [0]
   3. Don’t know [0]
2. **Why do the nurses test the blood? [maximum score: 3]**
3. To make sure that I and the baby have enough red blood cells to keep us healthy [0]
4. To make sure that I and my partner do not have a sexually transmitted disease [1]
5. To test for sugar [0]
6. To test for HIV/AIDS [1]
7. To test for cholesterol [0]
8. To test your blood type – if negative baby will need medication after birth [1]
9. Don’t Know [0]
10. **Should you ask for the results of your pap smear? [maximum score: 1]**
    1. Yes [1]
    2. No the clinic will contact you [0]
    3. Don’t know [0]
11. **Why do the nurses test the urine and blood pressure every visit? [maximum score: 1]**
12. To be sure my baby does not have a cleft palate [0]
13. To check for a serious complication called pre-eclampsia [1]
14. Don’t know [0]
15. **How can you stay healthy during pregnancy?** **[maximum score: 4]**
16. Have a drink if you feel anxious about your pregnancy [0]
17. Stop smoking [1]
18. Eat lots of fatty food to make sure your baby grows big and strong [0]
19. Gain at least 20 kilos to have a big healthy baby
20. Eat a healthy, balanced diet [1]
21. Don’t move around. Exercise is not good for your baby [0]
22. Don’t take any drugs [1]
23. All medicines are safe [0]
24. Only take medicine prescribed by the nurse or midwife [1]
25. Don’t know [0]
26. **Why should you take folic acid (from the clinic) during pregnancy? [maximum score: 1]**
27. It prevents early labour [0]
28. It decreases the risk of serious birth defects [1]
29. Too much folic acid can harm your baby [0]
30. Don’t know [0]
31. **How do drugs and alcohol affect the baby growing in the womb? [maximum score: 2]**
32. They don’t affect the baby [0]
33. During the first 12 weeks drugs and alcohol can cause major abnormalities with baby [1]
34. Even after the first 12 week the baby is still at risk for abnormalities [1]
35. Don’t know [0]
36. **Should you seek medical help outside your appointments? [maximum score: 2]**
37. No. The sisters will check everything that needs to be checked [0]
38. Yes. If you experience any abnormal symptoms such as bleeding, persistent frontal headache, sudden swelling of hands, feet and face seek medical help immediately [1]
39. Yes if the baby does not move in 12 hours [1]
40. Yes if I am tired [0]
41. Don’t know [0]
42. **What are the signs of labour? [maximum score: 3]**
43. When you have three contractions within 10 minutes [1]
44. Your water breaks [1]
45. Having to go to the toilet a lot [0]
46. Bleeding [1]
47. Vomiting [0]
48. Feeling the baby kick a lot [0]
49. Don’t know [0]
50. **Have you received sms’es about pregnancy? Please choose one answer**
    1. Yes
    2. No
    3. Not sure

*Only continue with the questions below for those who answer yes to question 10.*

1. **Did you miss more than two clinic appointments? Choose one answer.**
   1. Yes
   2. No
   3. Don’t know/Not sure
2. **Did you make sure you got the results of your pap smear? Choose one answer.**
3. Yes
4. No
5. Don’t know/Nor sure
6. N/A (for those who did not have a pap smear, i.e. women below 30 who are HIV-negative).
7. **Did you take folic acid and iron during your pregnancy? Please choose one answer.**
8. Yes
9. No
10. Don’t know/Not sure
11. **Did you drink alcohol during your pregnancy? Please choose one answer.**
12. Yes
13. No
14. Don’t know/Not sure
15. **Did you take drugs (such as tik, dagga, mandrax, heroin, cocaine etc.) during your pregnancy? Please choose one answer.**
16. Yes
17. No
18. Don’t know/Not sure
19. **Did you smoke (tobacco) during your pregnancy? Please choose one answer.**
20. Yes
21. No
22. Don’t know/Not sure
23. **Did you eat healthily during your pregnancy? Please choose one answer.**
24. Yes
25. No
26. Don’t know/Not sure
27. **Did you exercise/made sure you stayed fit during your pregnancy? Choose one answer.**
28. Yes
29. No
30. Don’t know/Not sure
31. **Did you take any prescription medication without discussing it with your health care provider during your pregnancy?**
32. Yes
33. No
34. Don’t know/Not sure
35. **From where did you get information about pregnancy? Choose as many answers as you like.**
36. Doctor/nurses/health promoters
37. TV/radio
38. Written material
39. SMSes
40. Friends/family/colleagues
41. Other
42. Did not get any new information
43. **Which source of information had most impact on you? Choose one.**
44. Doctor/nurses/health promoters
45. TV/radio
46. Written material
47. SMSes
48. Friends/family/colleagues
49. Other
50. Did not get any new information
51. **Did you find the SMSes useful? Choose one answer**
52. Yes
53. No
54. Don’t know/Not sure
55. **Did the SMSes give you new information that made you change your lifestyle or manage your pregnancy differently? Choose one answer.**
56. Yes
57. No
58. Don’t know/Not sure
