# Supplementary material for: Antenatal health promotion via short message service at a Midwife Obstetrics Unit in South Africa: a mixed methods study
Source: BMC Pregnancy Childbirth. 2014 Aug 21;14:284. doi: 10.1186/1471-2393-14-284 (PMC4158091; doi:10.1186/1471-2393-14-284)
Supplement: Supplementary file 4 — Additional file 4: Exit questionnaire. (DOCX 20 KB) [file 12884_2014_1164_MOESM4_ESM.docx]

**Focus group – guidelines/questions**

*Overall aim of focus group: Explore results from questionnaire*

1. Knowledge did not change substantially, yet high self-reported change in behaviour (due to SMSes). This could in some instances be due to the possibility that some women had high knowledge at baseline regarding no smoking, no alcohol, no drugs, etc.
2. Many indicated that SMSes had the most impact on them.
3. Many SMSes were not received: Is that because cell-phones were not charged, numbers changed, or phones were stolen? Was there sharing of information?
4. Also need to check how SMSes worked in relation to behaviour change theories (phases of change etc.) Could an explanation of behaviour change be that SMSes are not a good method to convey new information, improve knowledge, but works well as ‘cues to action’, motivating behaviour change?
5. Is it possible that they did change behaviour without knowledge change? (obviously a possibility where knowledge already high at baseline and it is possible that SMSes acted as cues to change.

*Theme 1: Why did knowledge not change significantly?*

1. Did you like the SMSes? Why/why not?
2. Do you think other people like them? Why/why not?
3. How many SMSes did you read? Why so few/many?
4. Do you think people read all of them?
5. How did the SMSes make you feel/how did you react when you received them?

Probe (anticipation, anxiety, irritation, motivation to change behaviour, started to think about changing). Perhaps use examples to indicate how people may react.

1. Do you know how other people reacted?
2. Did your reaction change over time?
3. Did you understand all the SMSes? Give an example – one of the more difficult SMSes. Probe.
4. Did they SMS campaign give you all the knowledge you felt you need? What were the gaps? What should they have said?

*Theme 2: Exploring behaviour change*: Why did they report behaviour change (despite no knowledge change)? Did they really change? Did they want to change/did they contemplate changing? Did they feel they had to say they changed (social desirability)?

1. Read out a knowledge SMS and a behaviour prompting SMS – what did you like most? Why? Which one is easiest to remember?
2. How did SMSes that told you to do something (give an example) – live healthily, not drink, not smoke etc. make you feel? (Irritated? Motivated? Excited? Couldn’t be bothered? Anxious – because didn’t feel I could do the thing, ambivalence, more easy to remember.)
3. Did you feel that you were back at school? Told what to do? (Did it create resistance?)
4. If you could say that SMSes had a ‘voice’ whose would it be - ‘the sister at the clinic’, your mother, your partner, a friend.
5. Did you change your lifestyle because of SMSes? If yes, what did you change? Was it easy? What made it easy? What made you do it? If no, why not?
6. Did you prefer the SMSes compared to receiving SMSes from the clinic, from pamphlets etc. Why/why not? Why are SMSes better?
7. Did the SMSes make you start thinking about changing behaviour/doing something differently? Why/why not?
8. Did some SMSes remind you about things you already knew and motivate you to do something about it? Give examples.
9. Change is not easy and many people tell you to do this and that. Did the SMSes make you feel that you had to change or say you changed even if you felt you could not? Do you think other people would react in that way?

*Theme 3: Cell phone use, SMSes and information sharing*

1. Did you share the information from SMSes with anybody? Whom? Why?
2. Did you share with other pregnant women/patients? Women at the clinic? (when sitting next to them, waiting, being bored?)
3. Do you share your cell phone with others (family, partner, friend?)? Is cell phone sharing common?
4. Did you change your phone/phone number during the SMS campaign?
5. Do you always have you cell phone with you, do you always have it on, charged?
6. Did you keep the SMSes or delete them. Why? Did you read them more than once?
7. How do you feel about the information you received on SMS compared to other sources, e.g. the information you received at the clinic? If different, why? (many indicated it is)
8. What would you do to make the SMSes better? (Are there too many, too much repetition, what about the tone, is it polite enough, is it patronizing, demanding). Would you like to receive ‘reminders’ later?
9. Create your own SMS - “how would it sound?” How would you like the ‘SMS person’ to ‘speak’?
10. Did you learn anything new from SMSes? What?
11. Something puzzles me: How come so many said they had change behaviour, but their knowledge had not changed much. Did you just say that because you had to?
